# Components Changes in Fresh Ginseng Pulp Treated With Commercial Sterilization and Its Potential Therapeutic in CTX‐Induced Liver Injury via Apoptosis and Nrf2‐MAPKs/NF‐κB Pathways

**DOI:** 10.1002/fsn3.71190

**Published:** 2025-11-17

**Authors:** Bo Nan, Guangquan Ren, Shuhan Ge, Yutong Liu, Linqing Zhou, Linlin Cui, Jing Ge, Yidan Luo, Haihua Shi, Xia Li, Yu Wang, Yuhua Wang

**Affiliations:** ^1^ College of Food Science and Engineering Jilin Agricultural University Changchun China; ^2^ Jilin Province Innovation Center for Food Biological Manufacture Jilin Agricultural University Changchun China; ^3^ National Processing Laboratory for Soybean Industry and Technology Changchun China; ^4^ National Engineering Research Center for Wheat and Cord Deep Processing Changchun China

**Keywords:** cyclophosphamide, ginseng, liver injury, oxidative stress, sterilization

## Abstract

Ginseng, as an important dietary supplement, possesses physiological activities including antitumor, immunoregulatory, and antioxidative effects. Ginsenosides are important components that play a physiological role, while minor ginsenosides exhibit stronger functions. Sterilization is the most effective method to increase the minor ginsenosides' contents, and it is also a prevalent technique in food processing. However, it is still unclear whether ginsenosides have protective effects on cyclophosphamide (CTX)‐induced liver injury, and the potential mechanism is still unclear. This research has found that sterilization treatment enhances the contents of total and minor ginsenosides, and also improves its antioxidant capacity, which is significant for the development of healthy food. CTX, a commonly used chemotherapy drug, has inevitable side effects, particularly serious damage to the liver. The purpose of this research is to investigate the improvement effects of fresh ginseng pulp (FGP) and sterilized ginseng pulp (SGP) on CTX‐induced liver injury in mice. Both FGP and SGP could reverse the increase of liver injury biomarkers induced by CTX, and also improve the equilibrium of essential antioxidant enzymes within the redox system, reducing the malondialdehyde (MDA) level. At the same time, FGP and SGP alleviated CTX‐induced liver oxidative damage by inhibiting the MAPKs, Nf‐κB signaling pathways and apoptosis, while enhancing the Nrf2/HO‐1/NQO1 antioxidant defense system. More importantly, SGP exhibits a superior effect in alleviating CTX‐induced liver injury. Cumulatively, SGP has the potential to become a functional food ingredient for alleviating the side effects of CTX‐induced liver injury, offering an important theoretical foundation for its development.

## Graphical abstract

SGP alleviated CTX‐induced liver injury via apoptosis and Nrf2‐MAPKs/NF‐κB pathways.

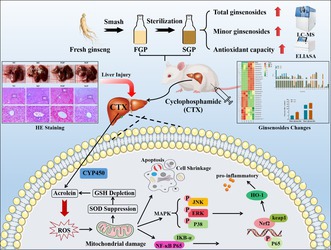

**Abbreviations**
ALT: alanine aminotransferase
AST: aspartate aminotransferase
BAX: Bcl‐2‐associated X protein
Bcl2: B‐cell lymphoma 2
CAT: Catalase
CTX: Cyclophosphamide
FGP: Fresh ginseng pulp
GSH: Glutathione
GSH‐Px: Glutathione Peroxidase
LPS: lipopolysaccharide
MAPKs: mitogen‐activated protein kinases
MDA: malondialdehyde
NF‐κB: nuclear factor kappa‐B
Nrf2: nuclear factor erythroid 2‐related factor 2
SGP: Sterilized ginseng pulp
SOD: Superoxide Dismutase
TNF‐α: tumor necrosis factor‐α

## 1 Introduction

Ginseng ( *Panax ginseng* C. A. Meyer), as an important new resource food, includes many active substances such as ginsenosides (Kim, Yoon, et al. 2024), polysaccharides (X. Zhang et al. 2024) and amino acids (Chen et al. 2015), which are widely utilized in traditional medicine, functional food and other industries due to its antitumor, antioxidative, antiinflammatory and antiaging properties (Dai, Liu, Ji, & Dai et al. 2025; Sun et al. 2025).

Generally, fresh ginseng is seldom used directly. In order to facilitate storage and commercial production, fresh ginseng is typically heat‐treated, which is helpful to extend the longevity of ginseng (H. Zhang et al. 2020). Heat treatment is the most widely used method for preserving and extending the longevity of food and nutritional supplements (J. Liu et al. 2023). This treatment is used to enhance the biological activity and increase the ginsenosides content of ginseng (Shin‐Jung et al. 2013). Heat treatment is frequently employed to process ginseng products, such as red ginseng and black ginseng. Notably, heat‐treated ginseng exhibits better bioactivity than fresh ginseng, with an increase in the variety and content of functional components (Zhang et al. 2023). Currently, scholars both within the country and abroad have conducted extensive research on the thermal processing of ginseng, focusing primarily on the chemical composition and physiological activity of ginseng following heat treatment. The ginsenosides solution was heated in a water bath to form the corresponding C‐20 deglycosylated secondary ginsenosides, thereby increasing the recovery rate of C‐20 deglycosylated ginsenosides and enhancing the content of minor ginsenosides through heat treatment. The composition of ginsenosides is significantly changed under heat treatment (such as cooking and drying), and protopanaxadiol ginsenosides (such as Rb1 and Rb2) and protopanaxatriol ginsenosides (such as Re and Rg1) will be degraded to produce minor ginsenosides (such as Rg2 and Rg3) (Yao et al. 2021). It has been confirmed that ginseng was heat‐treated at 90°C, 110°C, 130°C, and 150°C for 2 h, resulting in the improvement of the ginseng biological activity and formation of minor ginsenosides F2, F4, Rk3, Rh4, Rg3 (S‐type), Rg3 (R‐type), Rk1, and Rg5, which were not present in fresh ginseng (Hwang et al. 2014). Therefore, optimizing the conditions of heat treatment can not only regulate the composition of ginsenosides but also enhance the biological activity (Kang et al. 2006). This laid the foundation for processing fresh ginseng as a food ingredient.

Cancer is one of the major diseases threatening human health globally, with its incidence and mortality rates increasing annually (Cao et al. 2025). In the treatment of cancer, chemotherapy remains one of the significant treatment modalities, and cyclophosphamide (CTX), as a classic alkylating agent chemotherapy drug, has been extensively utilized in the treatment of various cancers since the 1950s (J. Li et al. 2022). It suppresses the proliferation of cancer cells by disrupting the DNA structure of tumor cells; however, it may also harm normal cells, resulting in adverse effects, which increase the burden on cancer patients (Osen et al. 2023). The liver is the primary metabolic organ of the body, involved in the secretion, storage, and detoxification of harmful substances. CTX is a chemotherapeutic drug that undergoes metabolism in the liver. Upon entering the body, CTX is transformed into active metabolites by liver microsomal enzymes, including acrolein and phosphoramide mustard (Jiang, Zhang, et al. 2020). Acrolein readily disrupts the organism's antioxidant defense system, leading to a significant imbalance in the oxidation‐antioxidant equilibrium. This ultimately results in the production of numerous free radicals, causing oxidative stress and thereby damaging the liver and other organs (Singh et al. 2018). This certainly does not exacerbate the suffering of cancer patients. Therefore, it is crucial to find a natural, nontoxic dietary supplement to alleviate liver damage caused by CTX.

High‐temperature and high‐pressure sterilization, which involves heating to 121°C for 30 min, is a widely used method in food processing. It effectively eliminates microorganisms and their spores, ensuring food safety and extending shelf life. The preliminary study confirmed the effects of heat treatment on the nonginsenoside functional components of fresh ginseng, and found that heat treatment significantly increased the content of substances with stronger biological activity (Zhang et al. 2023). However, the mechanism by which heat treatment of fresh ginseng improves CTX‐induced liver injury remains unclear. In this study, the effects of heat treatment on the ginsenosides content of fresh ginseng were investigated using targeted metabonomics technology, and the antioxidant capacity of fresh ginseng was assessed. On this basis, the protective mechanism of heat treatment against CTX‐induced liver injury was further clarified. This study provides a theoretical foundation and reference value for the development of ginseng health food with the potential to mitigate chemotherapy damage, and it is of significant importance for advancing the high‐quality development of the ginseng industry.

## 2 Materials and Methods

### 2.1 Material and Reagents

The fresh ginseng utilized in this experiment is 5‐year‐old, artificially cultivated ginseng, procured from Jingyu County (Jilin, China). CTX was obtained from Shanghai Yuanye Bio‐Technology Co. Ltd. (Shanghai, China). The alanine aminotransferase (ALT), aspartate aminotransferase (AST), catalase (CAT), total superoxide dismutase (T‐SOD), glutathione peroxidase (GSH‐Px) and malondialdehyde (MDA) were procured from Nanjing Jiancheng Bioengineering Institute (Nanjing, China). The glutathione (GSH) assay kit was purchased from Solarbio Science & Technology Co. Ltd. (Beijing, China). The antioxidant assay kits of ABTS^+^ and DPPH were purchased from Sigma‐Aldrich Corporation (St Louis, Missouri, USA). The ·OH assay kit was purchased from Nanjing Jiancheng Bioengineering Institute (Nanjing, China). The Fe^3+^ reducing ability detection reagent was purchased from Beijing Chemical Industry Group Co. Ltd. (Beijing, China). The primary antibodies Bcl‐2, Bax, Caspase‐3, p38, p‐p38, Nrf2 were supplied by Cell Signaling Technology (MA, USA), and the ERK, p‐ERK, JNK, p‐JNK, NQO1, HO‐1 were supplied by Wanlei Life Sciences Co. Ltd. (Shenyang, China). The secondary antibody *β*‐actin was supplied by ABclonal Technology Co. Ltd. (Wuhan, China). The other reagents used were of analytical grade and were sourced from standard commercial suppliers.

### 2.2 Preparation of Fresh Ginseng Pulp and Sterilized Ginseng Pulp

Select fresh ginseng with uniform size and thickness, ensuring it is free from mildew and has a good appearance. The fresh ginseng should be washed with tap water 3–5 times and then dried. Subsequently, the cleaned ginseng should be mixed with purified water at a 1:1 ratio and ground to a completely homogeneous state using a high‐speed blender set at 20,000 rpm for 2 min. Finally, fresh ginseng pulp was obtained (FGP). The fresh ginseng pulp was sterilized at 121°C for 30 min to obtain sterilized ginseng pulp (SGP).

### 2.3 Widely Targeted Analysis of Ginseng Functional Substances by LC–MS

50 mg ginseng pulp was placed in a 2 mL centrifuge tube, and the 600 μL 100% methanol internal standard extract was added and vortexed for 3 min. After 10 min centrifugation (12,000 rpm, 4°C), the supernatant was collected. The data acquisition instrument system primarily consists of ultra‐high‐performance liquid chromatography and tandem mass spectrometry. The chromatographic column employed is an Agilent SB‐C18 column (1.8 μm, 2.1 mm × 100 mm). The mobile phase A consists of ultrapure water (containing 0.1% formic acid), and the mobile phase B is composed of acetonitrile (containing 0.1% formic acid). The elution gradient is as follows: the proportion of phase B starts at 5% at 0.00 min, linearly increases to 95% by 9.00 min, remains at 95% for 1 min, then decreases back to 5%. The system is then equilibrated at 5% for 14 min, with a flow rate of 0.35 mL/min and a column temperature of 40°C. The injection volume is 4 μL. The conditions for mass spectrometry are as follows. The LIT and QQQ scans were obtained using the triple quadrupole linear ion trap mass spectrometer (Q TRAP) and the AB4500 Q TRAP UPLC/MS/MS system. The operating parameters of the ESI source are as follows: ion source, turbine spray; source temperature, 550°C; ion spray voltage (IS) 5500 V (positive ion mode)/‐4500 V (negative ion mode); ion source gas I (GSI), gas II (GSII), and curtain gas (CUR) were set to 50, 60, and 25.0 psi, respectively. In QQ and LIT modes, instrument tuning and quality calibration were performed using 10 and 100 μmol/L polypropylene glycol solutions, respectively. QQQ scanning utilizes Multiple Reaction Monitoring (MRM) mode, with the collision gas (nitrogen) set to a medium level. Through further optimization of the DP and CE, the DP and CE for each MRM ion pair are completed. Based on the metabolites eluted during each period, a set of specific MRM ions were monitored for each period, and the corresponding data was processed using the MWDB database.

### 2.4 Targeted Analysis of Ginsenosides by LC–MS

A 10 mg sample of ginseng was added to 200 μL of 50% methanol water to dissolve it. The mixture was then vortexed for 60 s, centrifuged at 4°C and 17,000 rpm for 15 min, after which the supernatant was taken for computer detection. Ultra‐high‐pressure liquid chromatography (UltiMate 3000) and high‐resolution mass spectrometry (X500R QTOF) equipment were utilized. The chromatographic column employed was an Acquity UPLC HSS T3 (1.8 μm, 2.1 × 100 mm). The mobile phase conditions were as follows: the column temperature was set at 40°C, the sample loading volume was 3 μL, with mobile phase A consisting of 0.1% formic acid in water and mobile phase B consisting of 0.1% formic acid in acetonitrile. Gradient elution was performed. The mass spectrometry conditions are as follows: the AB X500R Triple TOF mass spectrometer collects primary and secondary mass spectrometry data using the IDA function. During each data collection cycle, molecular ions with an intensity greater than 100 were screened out to collect the corresponding secondary mass spectrometry data. The parameters of the ESI ion source are set as follows: atomization pressure (GS1) at 60 Psi, auxiliary pressure at 60 Psi, air curtain pressure at 35 Psi, temperature at 650°C, and spray voltage at 5000 V in positive ion mode. PeakView software was used for quantitative analysis.

### 2.5 Animals and Experimental Design

The male specific pathogen‐free (SPF) BALB/c mice (5–6 weeks old, 20 ± 2.0 g) were purchased from Liaoning Changsheng Biotechnology Co. Ltd. [Liaoning, China, Certificate number: SCXK (Liao) 2020–0001]. The animal procedures were carried out in strict accordance with the guidelines of the Ethics Committee of the Laboratory Animal Center of Jilin Agricultural University (Animal Review No. 2019 04 10,005). The mice were allowed to acclimatize to the controlled conditions (25°C ± 1°C, 40%–50% relative humidity, 12 h light/dark cycle, free access to food and water) for 7 days. All experimental procedures involving animals and care were approved by the Jilin Agricultural University Animal Care Review Committee. The mice were randomized into four groups: NC (normal control group), MC (40 mg/kg/d CTX model group), FGP (40 mg/kg/d CTX +450 mg/kg/d fresh ginseng pulp) and SGP (40 mg/kg/d CTX +450 mg/kg/d sterilized ginseng pulp). The dosage of ginseng pulp (450 mg/kg/d) corresponds to our previous study (Bo et al. 2018). While the NC group was intraperitoneally (i.p.) injected with saline, the other groups were i.p. injected with 40 mg/kg body weight (BW)/d CTX (diluted in saline) for 3 days, as described previously (X. Jiang et al. 2019). In the 10 days after modeling, the FGP and SGP group mice were administered orally with ginseng pulp, and the NC and MC groups were given the same dose of sterilized water. The animal experimental design and procedure are shown Figure 2A. The mice were anesthetized for euthanasia after fasting for 12 h. The blood samples were obtained from the inner canthus and centrifuged (3000 rpm, 4°C, 20 min) to obtain the serum for biochemical analysis.

### 2.6 Organ Index and Histological Analysis

During the experiments, the mice body and organ weight data were recorded daily. The organ index was calculated as follows:

$$\text{Organ index}=\frac{\text{organ weight}\;\left(\mathrm{mg}\right)}{\text{body weight}\;\left(g\right)}$$

The liver tissue was fixed with 4% paraformaldehyde (Absin, Shanghai, China) for more than 24 h at room temperature, embedded in paraffin. The serial 5‐μm‐thick sections were obtained and utilized for hematoxylin and eosin (H&E) staining. The histological evaluation of liver tissue was observed by microscope (Leica, Germany) and the images were captured.

### 2.7 Biochemical Analysis

The liver tissue was homogenized (10%, w/v) in ice‐cold 50 mM phosphate buffer (pH 7.4), centrifuged (2500 rpm, 4°C, 10 min) and the supernatant was utilized to assay the physiological and biochemical levels. The levels of ALT, AST, MDA, GSH, GSH‐Px, T‐SOD, and CAT in the liver or serum were tested using commercial assay kits. All data were measured by a multimode plate reader (PerkinElmer, USA).

### 2.8 Western Blot Analysis

The liver tissue was lysed on ice for 20 min using a lysis buffer consisting of radio‐immunoprecipitation assay (RIPA) buffer, phenylmethanesulfonyl fluoride (PMSF) proteinase inhibitor and phosphatase inhibitor at a ratio of 98:1:1 (v/v/v). The liver tissue lysate was centrifuged (12,000 rpm, 4°C, 20 min) to obtain supernatant. The protein concentration of each homogenate supernatant was quantified using the BCA Protein Quantification Assay Kit (Beyotime, Shanghai, China). Generally, equal amounts of protein were separated by different concentrations of sodium dodecyl sulfate‐polyacrylamide gel electrophoresis (SDS‐PAGE, 8%, 10% or 12%) and transferred onto polyvinylidene fluoride (PVDF) membranes. The QuickBlock Blocking Buffer (Beyotime, Shanghai, China) was subsequently used to block the PVDF membranes at room temperature for 15 min. After incubation with the corresponding primary antibodies and horseradish peroxidase‐conjugated secondary antibodies, the chemiluminescence (ECL) system was used to visualize the intended proteins by an image scanner (iBright CL1000, Thermo Fisher Scientific, USA). Protein levels were normalized to the corresponding β‐actin expression and the band intensities were quantified by Image‐J software (The National Institutes of Health, USA).

### 2.9 Statistical Analysis

All experiments were performed in triplicate, and the data were expressed as mean ± SD. The statistical analysis was performed using GraphPad Prism 8.0 (San Diego, CA, USA) with one‐way analysis of variance (ANOVA). Values of *p* < 0.05 were considered statistically significant.

## 3 Results

### 3.1 Total Component Analysis of Functional Substances in FGP and SGP

**FIGURE 1:**
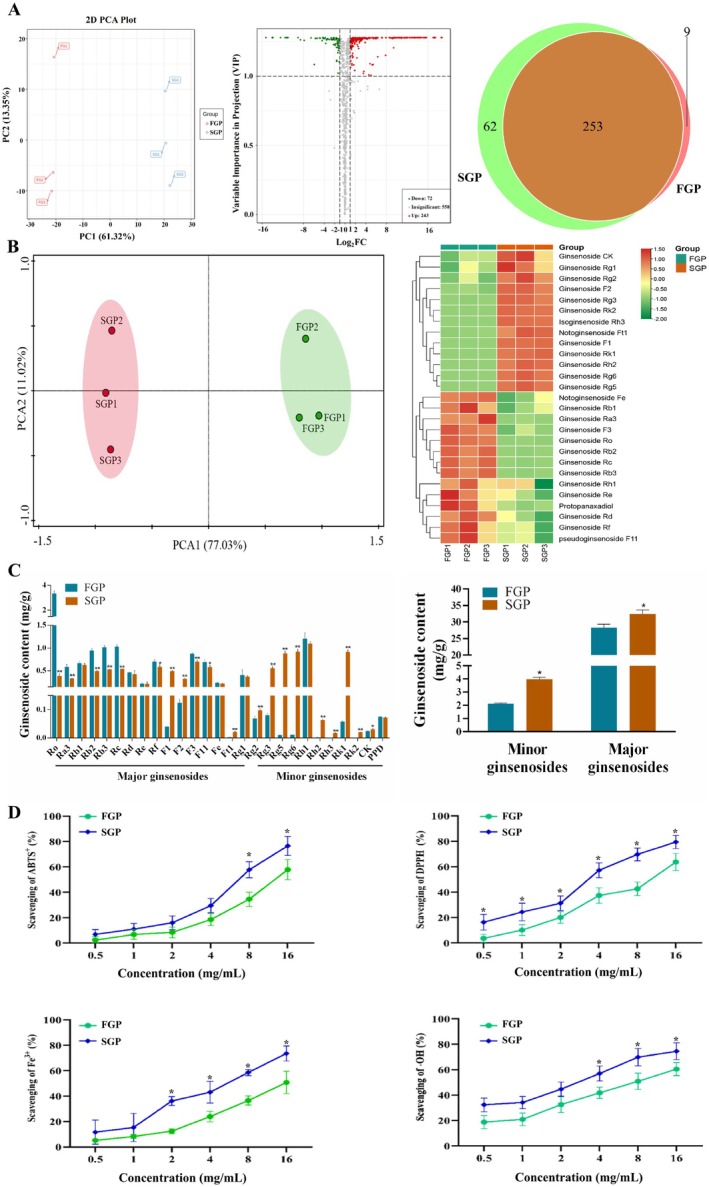
Effects of sterilization on functional substances, ginsenosides change, and antioxidant capacity in fresh ginseng pulp. (A) Functional substances PCA analysis, Volcano map, Venn diagram, (B) Ginsenosides change PCA analysis and cluster analysis, (C) Minor ginsenosides and total ginsenosides content, (D) Antioxidant capacity. *T*‐tests were used to statistically analyze multiple data sets. Compared with the FGP group, *p < 0.05.

In order to evaluate the compositional differences between FGP and SGP, a wide‐targeted metabonomics approach utilizing a UPLC‐ESI‐MS/MS system was employed to identify the distinct components present in each sample. The analysis of the QC‐TIC diagram indicated that the data exhibited excellent repeatability and reliability in this study. The PCA diagram clearly showed the distribution of principal components in two groups of ginseng pulp samples (Figure 1A). PC1 and PC2 were 61.32% and 13.53% respectively, indicating that the components in FGP were clearly separated from those in SGP. The volcano map illustrated the differences in composition between FGP and SGP (Figure 1A). As depicted on the volcanic map, red scattered dots signify a substantial increase in compounds, green dispersion points indicate a significant decrease in compounds, and gray scattered dots represent compounds with no significant difference. (VIP ≥ 1 and multiple change ≥ 2 or ≤ 0.5). There was no significant difference among 558 compounds, and among the 111 phenolic acids with significant differences, 243 compounds increased while 72 decreased. The Venn diagram illustrated the final screening results, with SGP samples denoted in red and FGP samples in green. Specifically, there are 9 unique compounds in FGP, 62 unique compounds in SGP, and FGP and SGP share 253 common compounds (Figure 1A). On the basis of clarifying the changes of functional substances in fresh ginseng pulp, the changes of ginsenosides were further analyzed.

### 3.2 Effects of Sterilization on Ginsenosides Change in Fresh Ginseng

The targeted metabolomics method, utilizing an LC–MS system, was employed to analyze the differences in ginsenosides between FGP and SGP. The PCA score plot indicated that PC1 accounted for 77.03% and PC2 for 11.02%, demonstrating that the ginsenosides in FGP were clearly distinguishable from those in SGP (Figure 1B, Figure S1). The heatmap displays all the altered ginsenosides in FGP and SGP, revealing that 27 ginsenosides experienced significant changes following commercial sterilization (VIP > 1.0 and *p* < 0.05). Among the 27 ginsenosides, 13 ginsenosides were found to have an increase in SGP, including ginsenosides CK, Rg1, Rg2, F2, Rg3, Rk2, Rh3, F1, Rk1, Rh2, Rg6, Rg5, and notoginsenoside Ft1. In contrast, the ginsenosides Rb1, Ra3, F3, Ro, Rb2, Rc, Rb3, Rh1, Re, Rd., Rf, protopanaxadiol, notoginsenoside Fe, pseudoginsenoside F11 were decreased after sterilization treatment in ginseng pulp. The content of major ginsenosides, including ginsenosides Ra3, Rb1, Rb2, Rb3, Rc, Rd., Re, Rf, Rg1, Rg2, Rg6, F3, and Ro was decreased in SGP, whereas the content of the minor ginsenosides including ginsenosides Rg3, Rg5, F1, F2, Rh2, Rk1, and CK was increased after sterilization treatment. The total content of minor ginsenosides in FGP was 2.11 mg/g, while that in SGP was 3.98 mg/g, which significantly increased (*p* < 0.05) (Figure 1C, Table S1). Notably, ginsenosides Rk2 and Rh3, which were not exist in fresh ginseng, but obtained by sterilization and transformation, and the contents of ginsenosides Rk2 and Rh3 were 0.02 mg/g and 0.0175 mg/g respectively (Figure 1C, Table S1). Thus, sterilization increased the content of minor ginsenosides, promoted the transformation of major ginsenosides into minor ginsenosides, and subsequently enhanced the activity of ginseng.

### 3.3 Effects of Sterilization on Antioxidant Activity in Fresh Ginseng

To investigate the impact of sterilization on the antioxidant potential of fresh ginseng pulp, the ABTS^+^ scavenging rate, DPPH scavenging rate, Fe^3+^ reduction capacity, and ·OH scavenging rate were employed as assessment metrics. Both FGP and SGP exhibit the ability to scavenge ABTS^+^, and this capacity increases with rising sample concentration. At a concentration of 16 mg/mL, the scavenging rate of FGP against ABTS^+^ is 57.91%, whereas SGP is 76.60%. This indicates that SGP has a greater scavenging ability for ABTS^+^ compared to FGP, suggesting that both FGP and SGP exhibit significant scavenging abilities towards ABTS^+^. The scavenging ability of FGP and SGP towards DPPH is depicted in Figure 1D, indicating that a higher scavenging rate correlates with superior free radical scavenging capacity for both groups of samples. As illustrated in Figure 1D, both FGP and SGP exhibit scavenging ability towards DPPH, with the effect intensifying as the sample concentration increases. When the FGP and SGP concentration is 16 mg/mL, the scavenging rates for DPPH are 63.72% and 79.48%, respectively (*p* < 0.05), which indicates that both FGP and SGP can reduce the chain reaction effect of peroxide radical, alkyl radical or lipid radical. Compared with FGP, SGP has stronger DPPH scavenging ability, which indicates that the antioxidant activity of fresh ginseng is enhanced after sterilization. The total reduction capacity of FGP and SGP is depicted in Figure 1D. As the concentration of FGP and SGP increases, the total reduction capacity of both FGP and SGP also increases, demonstrating a good dose‐dependent relationship. When the concentration is 16 mg/mL, the reduction rates of Fe^3+^ are 50.79% and 73.61%, respectively (*p* < 0.05). Compared with SGP, FGP exhibits a stronger reduction capacity. Both FGP and SGP have the ability to scavenge ·OH, and with the increase of sample concentration, the ability to scavenge ·OH is also enhanced, reaching 60.43% and 74.47%, respectively, and SGP has a stronger ability to scavenge ·OH (*p* < 0.05). Therefore, to sum up, the antioxidant activity of SGP is enhanced after sterilization. This process significantly enhanced the functionality of ginseng, which laid a foundation for further exploring the protective mechanism of CTX‐induced liver injury.

### 3.4 Effects of FGP and SGP on Body Weight, Food Intake and Organ Indexs in CTX‐Induced Mice

**FIGURE 2:**
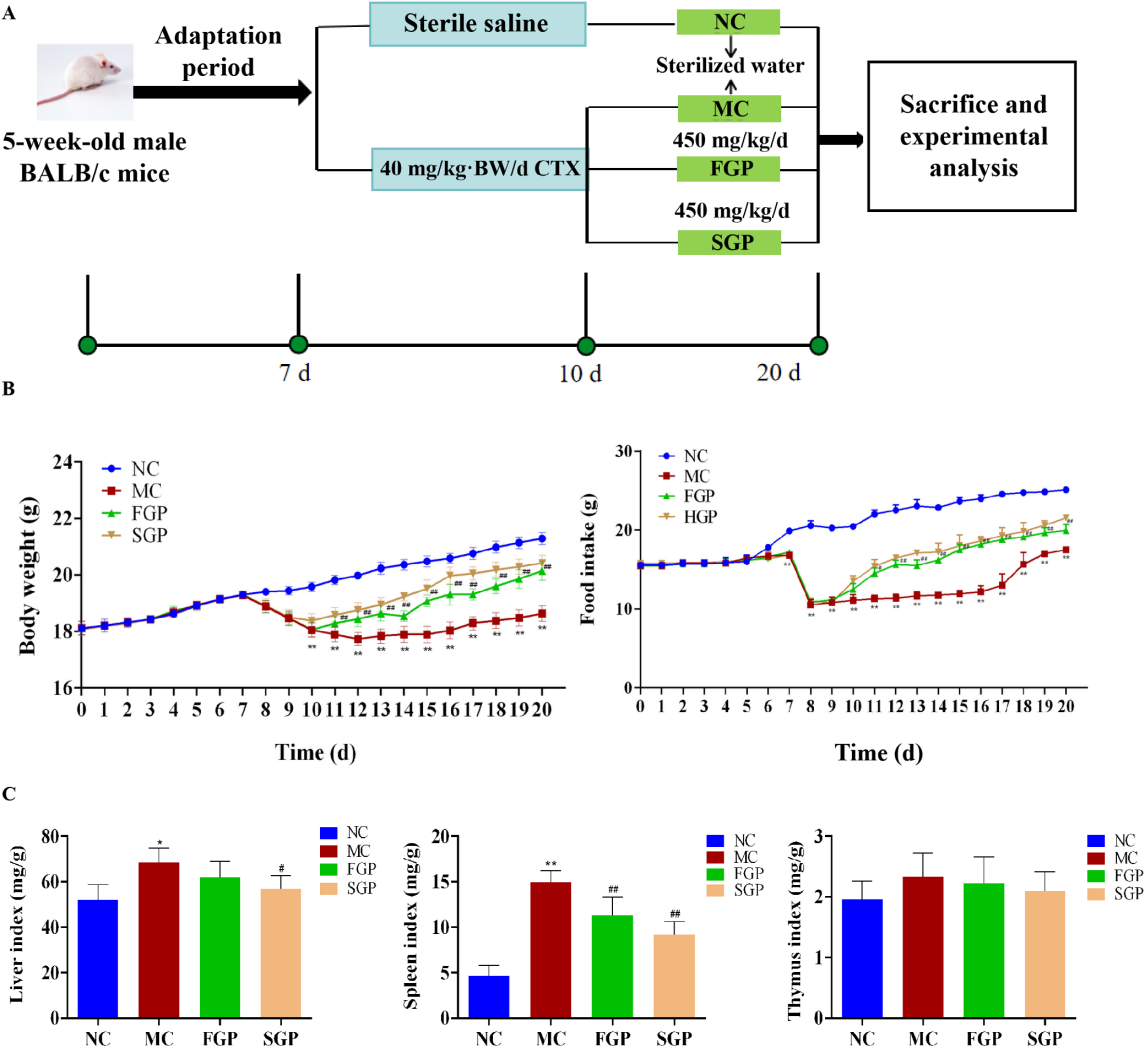
Experimental design of liver injury mice induced by CTX and the effects of FGP and SGP on body weight, food intake, and organ indexes in CTX‐induced mice. (A) Experimental design, (B) Body weight and food intake, (C) Organ indexes. The data were expressed as mean ± SD (*n* = 10). One‐way ANOVA was used to statistically analyze multiple data sets. Compared with the NC group, *p < 0.05, **p < 0.01. Compared with the MC group, #*p* < 0.05, ##*p* < 0.01.

The weight of mice is a crucial physiological indicator, reflecting not only their health status but also directly impacting the reliability and repeatability of experimental results. The mice in each group were in relatively good condition, and the weight of the mice in the NC group increased steadily. After 3 days of intraperitoneal injection of CTX, the weight of mice in the MC group, FGP group and SGP group decreased significantly. After FGP and SGP intervention, the weight of CTX‐induced mice in the FGP and SGP groups was improved (*p* < 0.01) (Figure 2B). Compared with the NC group, the food intake of mice in the MC group decreased significantly (*p* < 0.01), aligning with the symptoms of weight loss and anorexia observed in chemotherapy patients. Following the intervention of FGP and SGP, the food intake of mice increased significantly, indicating that FGP and SGP can alleviate the side effects caused by CTX (*p* < 0.01) (Figure 2B). The liver index is one of the important indexes to evaluate liver health and the degree of liver injury. Compared with MC group mice, the liver index of the SGP group decreased significantly (*p* < 0.05) (Figure 2C). The preliminary results indicate that both FGP and SGP interventions can mitigate mice liver injury caused by CTX, with SGP demonstrating a superior effect compared to FGP. The spleen index yielded similar results. CTX caused an increase in the spleen index in mice, whereas FGP and SGP significantly decreased this index following intervention (*p* < 0.01) (Figure 2C). However, there was no notable impact on the thymus index (*p* < 0.01) (Figure 2C). This suggests that FGP and SGP effectively improve liver and spleen injury in mice induced by CTX.

### 3.5 Effects of FGP and SGP on Liver Injury in CTX‐Induced Mice

**FIGURE 3:**
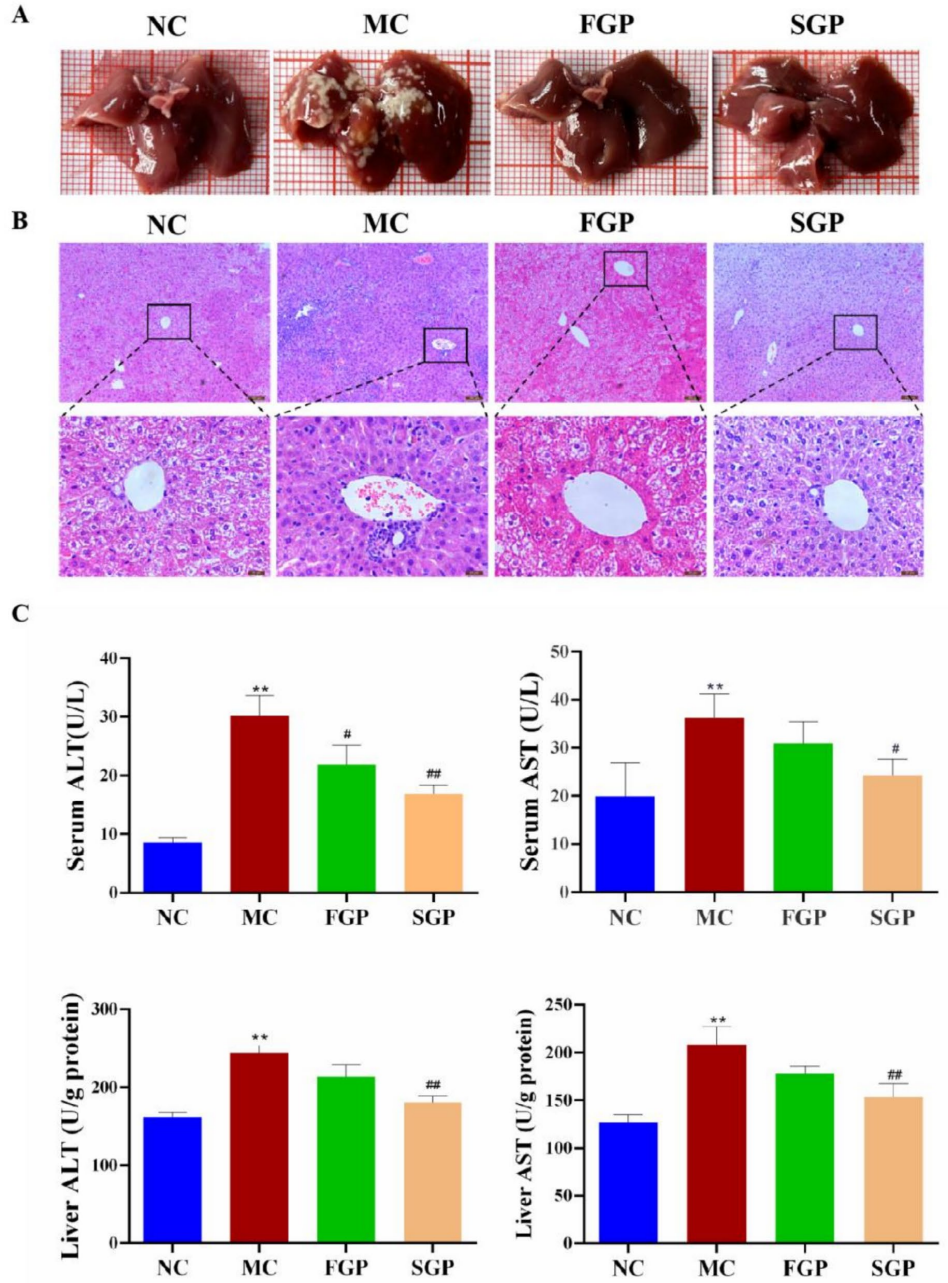
Effects of sterilized fresh ginseng pulp on liver injury in CTX‐induced mice. (A) Mice liver morphology, (B) H&E staining (400×), (C) Serum and liver transaminase (ALT/AST) levels. The data were expressed as mean ± SD. One‐way ANOVA was used to statistically analyze multiple data sets. Compared with the NC group, *p < 0.05, **p < 0.01. Compared with the MC group, #*p* < 0.05, ##*p* < 0.01.

Liver morphology is an indispensable index in the evaluation of liver injury. H&E staining revealed that the liver tissue of the NC group mice appeared normal, with intact hepatic lobules and sinuses. The hepatic cords were neatly arranged in a radial pattern, with distinct boundaries of hepatocytes and no observed inflammatory cell infiltration. In contrast, the liver tissue of MC group mice exhibited incomplete hepatic lobules, hepatocytes with varying degrees of swelling and disordered arrangement, disrupted hepatic cords, compromised nuclear structure of hepatocytes, and evident congestion of the central vein. Compared to the MC group, the hepatocytes of mice in the FGP and SGP groups were more neatly arranged, nuclei were clearly visible, and inflammatory cell infiltration was less pronounced. Consequently, FGP and SGP significantly ameliorate the morphological damage to the mice liver induced by CTX and play a protective role for the liver (Figure 3A,B).

ALT and AST are effective indexes to evaluate liver function. Compared with the NC group, the levels of ALT and AST in the serum and liver of MC group mice were significantly increased (*p* < 0.05), which indicated that the mice liver injury model induced by CTX was successfully established. After the intervention of FGP and SGP, the levels of AST and ALT in the serum and liver of mice recovered, and the levels of ALT and AST in the serum and liver of the SGP group were significantly lower than those of the MC group (Figure 3C). Therefore, both FGP and SGP can improve the liver injury induced by CTX in mice, and SGP has a better improvement effect.

### 3.6 Effects of FGP and SGP on Liver Oxidative Damage in CTX‐Induced Mice

**FIGURE 4:**
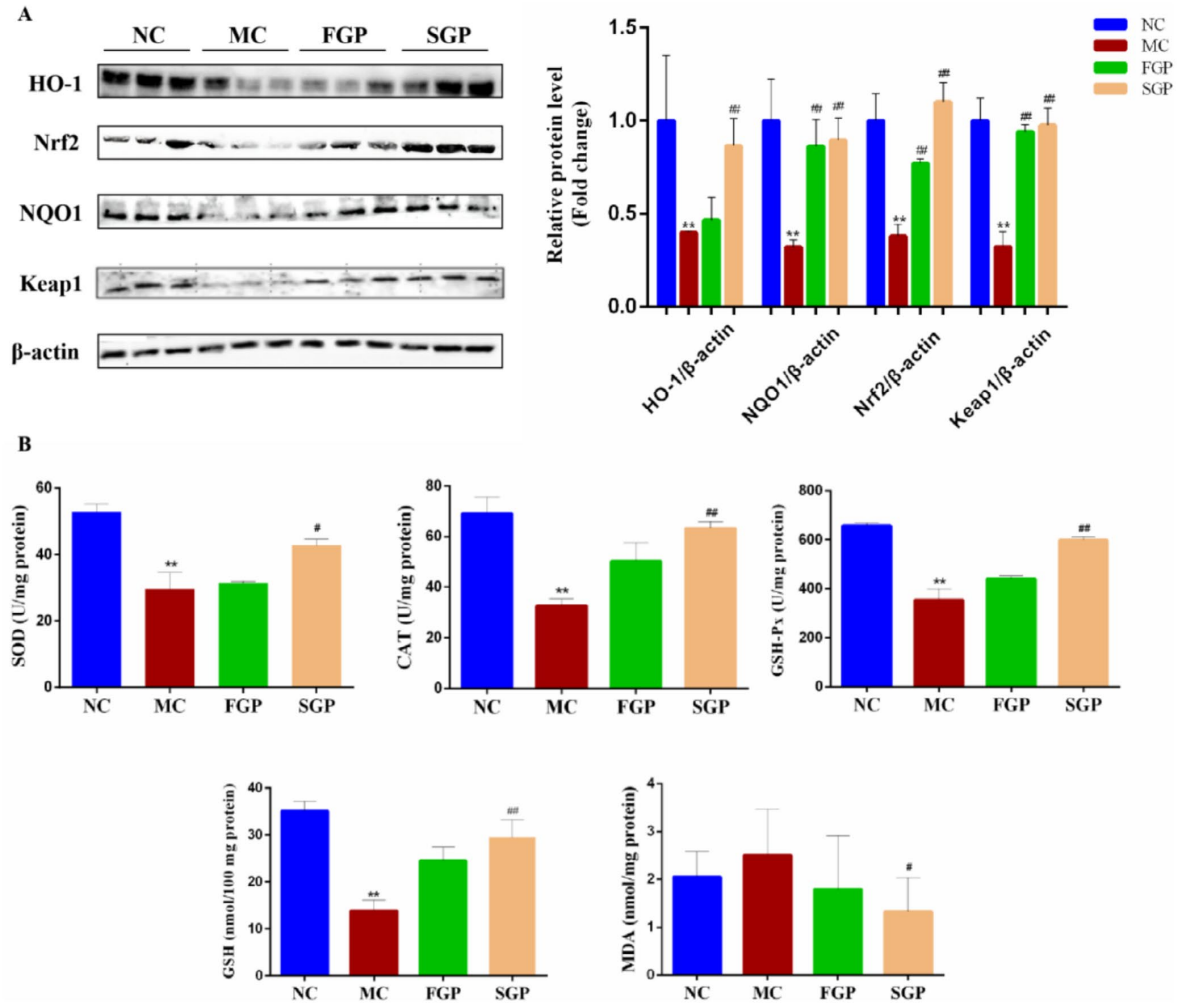
Effects of sterilized fresh ginseng pulp on oxidative damage in CTX‐induced mice. (A) The expression of Nrf2/HO‐1/NQO1 signaling pathway proteins, (B) Antioxidant enzyme system related indexes. The data were expressed as mean ± SD. One‐way ANOVA was used to statistically analyze multiple data sets. Compared with the NC group, **p < 0.01. Compared with the MC group, #*p* < 0.05, ##*p* < 0.01.

To investigate the effects of FGP and SGP on oxidative stress in the mice liver induced by CTX, the activity of antioxidant enzymes and the levels of glutathione (GSH) and malondialdehyde (MDA) were measured. GSH and MDA are key indicators of oxidative stress; the decrease of GSH level and the increase of MDA level reflect the aggravation of liver oxidative stress. Acrolein, a metabolic byproduct of CTX, binds with intracellular glutathione and consumes it, resulting in the impairment of the glutathione‐dependent antioxidant system and subsequent apoptosis (Hong et al. 2022). In the MC group, the GSH level was reduced (*p* < 0.01) and the MDA level was elevated compared to the NC group mice liver. Both liver GSH and MDA levels changes were restored in the FGP and SGP groups, and the SGP group showed a significant difference relative to the MC group (Figure 4B). Antioxidant enzymes, including Superoxide Dismutase (SOD), Catalase (CAT) and Glutathione Peroxidase (GSH‐Px), play crucial roles in maintaining cellular functional integrity by protecting cells from oxidative damage induced by superoxide anions (Lan et al. 2024). In this study, CTX significantly reduced the activities of SOD, CAT and GSH‐Px in the liver, suggesting that CTX damaged the antioxidant defense mechanism of the liver. In contrast, the activities of SOD, CAT and GSH‐Px were significantly increased in the liver after FGP and SGP intervention, and the protective effect of SGP was better than that of FGP (Figure 4B). Therefore, mitigating oxidative damage is an essential strategy for alleviating CTX‐induced liver injury.

To further investigate the mechanisms by which FGP and SGP protect against CTX‐induced liver injury in mice, the expression of proteins associated with the Nrf2/HO‐1/NQO1 signaling pathway was examined in the liver. The levels of HO‐1, NQO1, Nrf2 and Keap‐1 protein expression were significantly reduced in MC group mice liver (*p* < 0.01). Conversely, in the FGP and SGP group, FGP and SGP intervention significantly increased the protein expression compared to the MC group (*p* < 0.05) (Figure 4A). Therefore, FGP and SGP can maintain the redox homeostasis of the organism by regulating the expression levels of relevant proteins in the Nrf2/HO‐1/NQO1 signaling pathway. These findings indicated that FGP and SGP treatment alleviated the antioxidant capability in CTX‐induced mice liver.

### 3.7 Effects of FGP and SGP on Liver Inflammation in CTX‐Induced Mice

**FIGURE 5:**
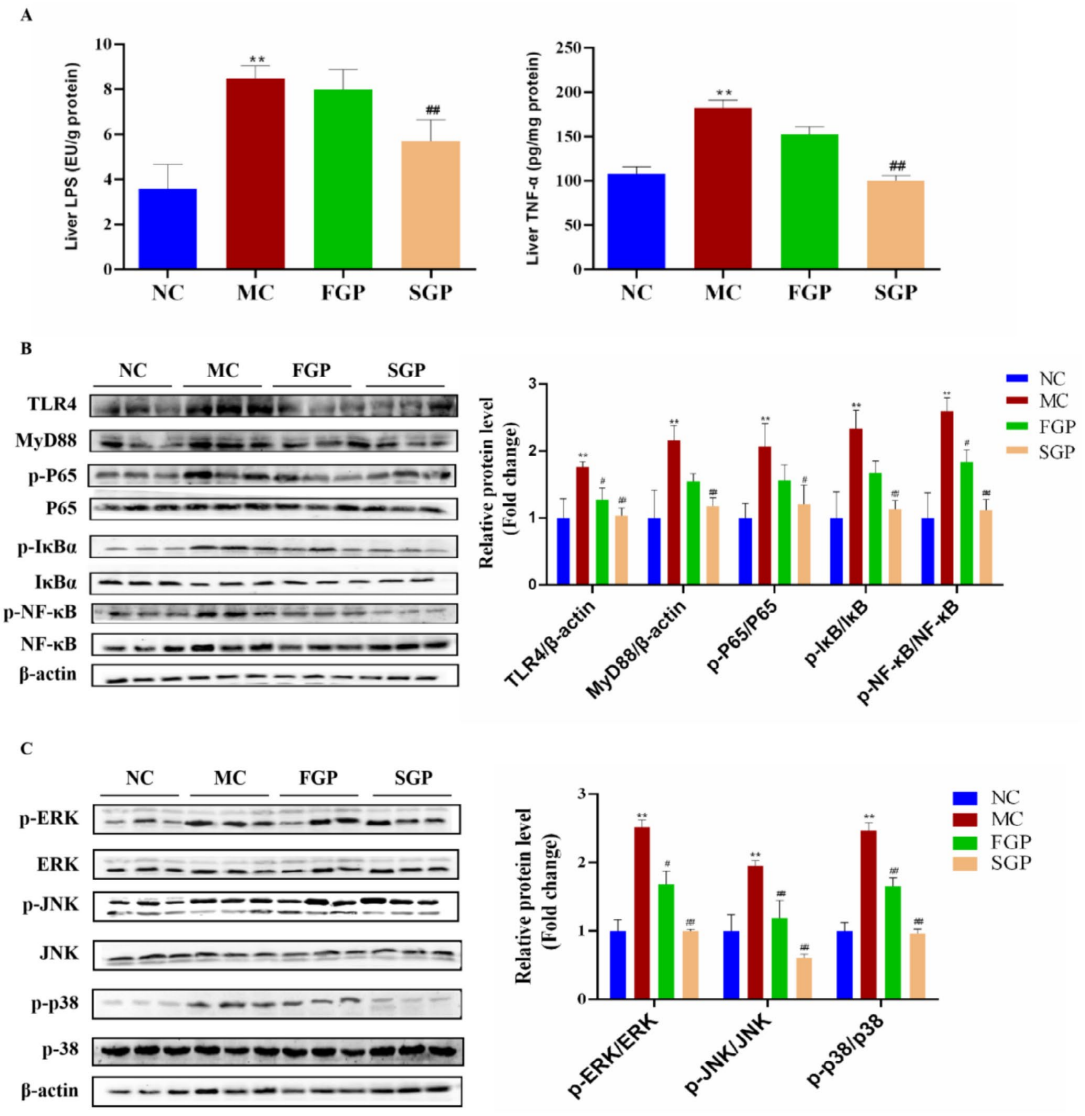
Effects of sterilized fresh ginseng pulp on liver inflammation in CTX‐induced mice. (A) The liver LPS and TNF‐α levels, (B) The expression of TLR4/NF‐κB signaling pathway proteins, (C) The expression of MAPK signaling pathway proteins. The data were expressed as mean ± SD. One‐way ANOVA was used to statistically analyze multiple data sets. Compared with the NC group, **p < 0.01. Compared with the MC group, #*p* < 0.05, ##*p* < 0.01.

Lipopolysaccharide (LPS) is the primary component of the outer membrane of Gram‐negative bacteria, which is closely associated with inflammatory reactions and affects the production of both antiinflammatory and proinflammatory cytokines, particularly tumor necrosis factor‐α (TNF‐α). In this research, CTX induced a significant increase in the levels of LPS and TNF‐α in mice liver (*p* < 0.01), and the intervention of FGP or SGP significantly inhibited the elevation of these proinflammatory cytokines (Figure 5A). Toll‐like receptor 4 (TLR4) is a pattern recognition receptor, and nuclear factor kappa‐B (NF‐κB) is an important transcription factor, which is involved in regulating the expression of inflammation‐related genes. LPS stimulation can activate the TLR4/NF‐κB signaling pathway and plays a key role in the process of cellular inflammatory response. Therefore, the expression levels of related proteins on the TLR4/NF‐κB signaling pathway were determined. Compared with the NC group, the TLR4, MyD88, p‐P65/P65, p‐IκBα/IκBα, and p‐NF‐κB/NF‐κB protein levels were markedly increased in MC group mice liver (*p* < 0.01). Oppositely, it was found that FGP and SGP intervention significantly inhibited the expression levels of TLR4, MyD88, p‐P65/P65, p‐IκBα/IκBα, and p‐NF‐κB/NF‐κB, suggesting that FGP and SGP attenuated CTX‐induced liver inflammation (Figure 5B). Mitogen‐activated protein kinase (MAPK) is a group of evolutionarily conserved serine/threonine protein kinases that is activated by a series of extracellular stimuli, including oxidative stress, and regulates many physiological activities, such as inflammation and apoptosis. As shown in Figure 5C, CTX activated the MAPK signaling pathway, resulting in a significant increase in the expression levels of p‐ERK, p‐JNK and p‐p38 proteins (*p* < 0.01). The phosphorylation levels of ERK, JNK and p38 were significantly inhibited in the FGP and SGP groups mice compared with the MC group (*p* < 0.05), suggesting that FGP and SGP ameliorated CTX‐induced liver inflammation by down‐regulating the MAPK signaling pathway.

### 3.8 Effects of FGP and SGP on Liver Apoptosis in CTX‐Induced Mice

**FIGURE 6:**
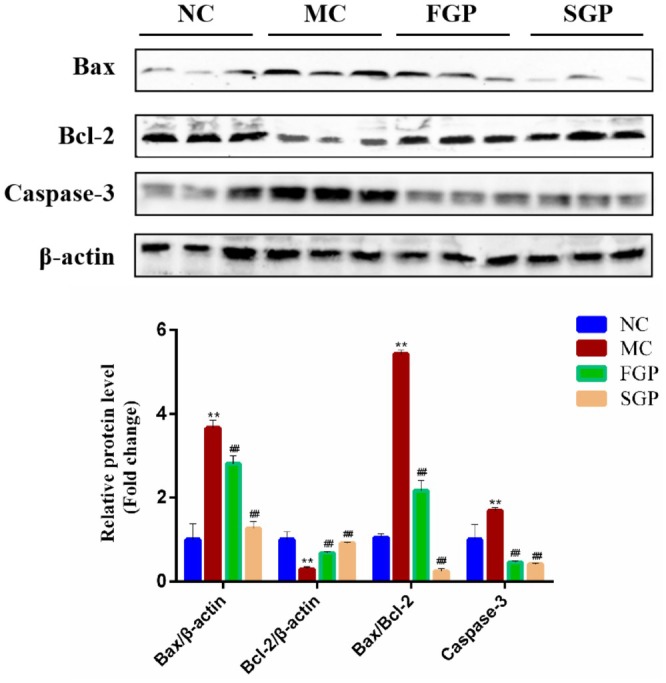
Effects of sterilized fresh ginseng pulp on liver apoptosis in CTX‐induced mice. The expression of apoptosis‐related proteins. The data were expressed as mean ± SD. One‐way ANOVA was used to statistically analyze multiple data sets. Compared with NC group, **p < 0.01. Compared with MC group, #*p* < 0.05, ##*p* < 0.01.

Apoptosis is not only an important mechanism of liver injury but also plays a key role in the occurrence, development, and treatment of liver disease. Inflammatory cytokines released after the activation of the TLR4/NF‐κB signaling pathway can induce apoptosis. In this study, CTX significantly increased the expression levels of the proapoptosis marker proteins Bax (*p* < 0.01) and Caspase‐3 (*p* < 0.05), and significantly decreased the expression of the antiapoptosis protein Bcl‐2 (*p* < 0.05) (Figure 6). In addition, CTX significantly increased the ratio of Bax/Bcl‐2 (*p* < 0.0001). In contrast, both FGP and SGP exerted antiapoptotic effects by significantly inhibiting the Bax/Bcl‐2 ratio and reducing Caspase‐3 protein levels, with SGP exhibiting superior antiapoptotic effects. This suggests that FGP and SGP efficiently attenuated CTX‐induced liver apoptosis.

## 4 Discussion

Ginseng, a popular and significant dietary supplement, exhibits a variety of functional activities such as antioxidation, antiinflammation, antifatigue, and immunity enhancement, and is widely utilized in health foods (Y. Y. Lee et al. 2015; Shin et al. 2018). In general, ginseng is primarily processed into white ginseng (air‐dried after harvest) and red ginseng (steamed or heat‐treated) to facilitate storage and enhance its biological activity (Huang et al. 2023). However, there are few studies on how food processing methods affect the ginsenosides in fresh ginseng. The heat treatment conditions utilized in this study not only adhere to the commercial sterilization process but also significantly enhance the content of minor ginsenosides. Subsequently, the mechanism by which sterilizing fresh ginseng alleviates CTX‐induced liver injury was further analyzed.

In this study, the sterilization treatment resulted in an increase in the content of minor ginsenosides in ginseng pulp, including ginsenosides Rg2, Rg3, Rg5, Rg6, Rh2, among others, and also enhanced the antioxidant capacity of ginseng. The core mechanism is that the chemical structure determines its biological activity. Minor ginsenosides are generally obtained through the degradation of major ginsenosides. Major ginsenosides, with large molecular weights, multiple glycosyl groups, and strong polarity, find it difficult to penetrate the cell membrane, leading to low bioavailability, and consequently, weak direct antioxidant capacity. Minor ginsenosides have smaller molecular weights and lower polarities, which facilitate easier absorption and allow for more effective interaction with free radicals or regulation of the intracellular antioxidant signaling pathway (J. Jin et al. 2006; Mengmeng et al. 2018; Wang et al. 2022). The study shows that ginsenoside Rg3 can stabilize the mitochondrial membrane potential, inhibit the release of cytochrome C, enhance the efficiency of the mitochondrial respiratory chain, reduce the “passive leakage” of ROS, and play an antioxidant role by protecting mitochondrial function (S. J. Lee et al. 2019). Ginsenoside Rg5, as an effective agonist of Nrf2, plays an antioxidant role by regulating the Nrf2 signaling pathway (Kim, Kim, et al. 2024). The increase in minor ginsenosides content is closely related to the enhancement of antioxidant capacity.

After CTX enters the body, it is primarily metabolized in the liver. The acrolein produced during this metabolic process further induces the generation and accumulation of ROS in the liver, leading to oxidative stress. Oxidative stress results in structural damage to hepatocyte organelles, which ultimately leads to inflammation and apoptosis of hepatocytes. It has been proven that the active components of ginseng, primarily ginsenosides, can directly scavenge free radicals by bolstering the organism's endogenous antioxidant defense system, thereby alleviating oxidative stress caused by CTX metabolites. This further inhibited the inflammatory response and apoptosis downstream of oxidative stress, ultimately protecting liver cells from damage (Abdelfattah‐Hassan et al. 2019; Zhu et al. 2015). ALT and AST levels are the key biomarkers to evaluate liver injury (Zhou et al. 2024). In this study, CTX induced a significant increase in mice liver (serum) ALT and AST levels, indicating that CTX caused liver damage in mice. CTX‐induced hepatotoxicity is also characterized by the disruption of the antioxidant defense system, which is the core mechanism responsible for counteracting oxidative stress. Key antioxidant enzymes, including SOD, CAT and GSH‐Px serve as critical biomarkers for assessing oxidative stress levels and antioxidant capacity. Acrolein depletes cellular GSH, leading to the impairment of the GSH‐dependent antioxidant defense mechanism and triggering inflammation and apoptosis. Therefore, inhibiting oxidative stress and regulating the antioxidant defense system are crucial approaches for alleviating CTX‐induced liver injury. MDA is the end product of lipid peroxidation, reflecting the exacerbation of oxidative stress and cell membrane damage (Jiang et al. 2022). When the MDA level is higher, it indicates that the tissue damage is more serious. In this study, CTX resulted in a decrease in the GSH level and the activities of CAT, SOD, and GSH‐Px enzymes in the mice liver, as well as an increase in the MDA level. However, this phenomenon was reversed to some extent following the supplementation of FGP and SGP, indicating that FGP and SGP can restore the oxidative balance in CTX‐induced liver injury. Nrf2 is a key regulator of cellular redox reactions, which inhibits the NF‐κB signaling pathway during oxidative stress and inflammatory responses (Jiang, Zhang, et al. 2020). The absence of Nrf2 leads to the activation of the NF‐κB signaling pathway, which promotes the excessive production of cytokines and inflammatory cells (C. Jin et al. 2024). Generally, Nrf2 binds to Keap‐1 to prevent nuclear translocation under normal circumstances. However, Nrf2 dissociates from Keap‐1, which induces the expression of the antioxidant enzyme system after external stimulation, thereby reducing inflammation, oxidative stress, and hepatocyte death. CTX induces a large number of intracellular ROS through its metabolites, phosphoramide mustard and acrolein, and the released ROS disrupts the interaction between Keap‐1 and Nrf2 via electrophilic modification, thereby activating the Nrf2 pathway. Our findings indicated that CTX suppresses the expression of Nrf2 and HO‐1 in the liver, thereby exacerbating CTX‐induced liver injury. More importantly, FGP and SGP increased the Nrf2 and HO‐1 expression in CTX‐induced mice liver. These results indicated that FGP and SGP can mitigate CTX‐induced liver injury by modulating oxidative stress.

LPS plays a role in the process of liver inflammation and oxidative stress, which is a significant contributor to liver injury (Li, Shi, et al. 2020). The permeability of intestinal mucosa is increased, and a large amount of LPS is released into the liver through the portal vein when the liver is damaged, which activates the TLR4/NF‐κB pathway and induces liver inflammation (C. Tao et al. 2023). The immune system promptly releases TNF‐α to initiate inflammation when the organism encounters an infection, sustains an injury, or is otherwise stimulated. In this study, as expected, the levels of LPS and TNF‐α were increased in CTX‐induced mice liver, and FGP and SGP could effectively improve the levels of inflammatory factors in the liver caused by CTX. LPS binds to TLR4 on the surface of liver immune cells, activates downstream NF‐κB and MAPK pathways via MyD88, and triggers the release of numerous proinflammatory factors. In summary, LPS has emerged as a critical hub for CTX‐induced liver injury, mediated by a cascade reaction involving “intestinal leakage, liver inflammation, oxidative damage, and liver injury.” Therefore, targeted alleviation of LPS‐induced inflammation is an important direction to alleviate liver injury. This study confirmed that FGP and SGP can inhibit the activation of the TLR4/NF‐κB pathway by down‐regulating the expression levels of TLR4, p‐IκBα, and p‐p65 proteins in the liver. Consequently, FGP and SGP can mitigate CTX‐induced liver injury by suppressing inflammation.

MAPK pathway induces transcription of target genes related to cell proliferation, survival and apoptosis when activated by inflammation (F. Tao et al. 2024). Among MAPK‐mediated signaling pathways, the p38 MAPK pathway is considered the most important as a key regulator of numerous cellular biological functions. The activation of p38 MAPK is closely related to inflammation and oxidative stress (Zhai et al. 2019). It can regulate numerous downstream transcription factors, including NF‐κB, up‐regulate the expression of inflammatory molecules, and promote the occurrence and development of inflammation. Oxidative stress, inflammation and apoptosis promote each other through complex signal networks, forming a vicious circle and promoting the development of liver diseases (L. Li, Chu, et al. 2020). Consequently, reducing oxidative stress and inflammation in the liver is crucial for maintaining liver health. In the current study, we found that FGP and SGP have a protective effect on CTX‐induced liver injury, mainly through antioxidation and the regulation of cellular inflammatory signaling pathways. The inflammatory mediators produced by inflammation can induce apoptosis in various ways. TNF‐α can bind to death receptors on the cell surface, activate the caspase cascade reaction and lead to cell apoptosis (Ding et al. 2024). Simultaneously, the Bax/Bcl‐2 signaling pathway plays a crucial role in the regulation of cellular apoptosis. The Bcl‐2 acts as a cell survival factor, whereas Bax facilitates cell death (Dogan et al. 2025; Kazmi et al. 2025). In this study, CTX significantly increased the expression levels of proapoptotic marker proteins Bax and Caspase‐3, and significantly decreased the expression of the antiapoptotic protein Bcl‐2. In contrast, FGP and SGP exerted antiapoptotic effects by significantly inhibiting the Bax/Bcl‐2 ratio and Caspase‐3 protein levels, suggesting that FGP and SGP efficiently attenuated CTX‐induced liver apoptosis. Therefore, our data provide a theoretical foundation for the comprehensive development of fresh ginseng. In summary, this study has demonstrated that ginseng exerts a protective effect against CTX‐induced liver injury by improving oxidative stress, reducing inflammation, and modulating the apoptosis signaling pathway. However, the study did not include a positive control group; we only analyzed the effects of FGP and SGP on CTX‐induced liver injury. Studies have indicated that ginseng, acting as a hepatoprotective agent, enhances the liver's resistance to CTX‐induced injury. It accomplishes this by mitigating oxidative stress, inflammation, and apoptosis. Notably, its efficacy surpasses that of vitamin E, which serves as the positive control in these studies (Abdelfattah‐Hassan et al. 2019). Therefore, to further illustrate the problem, we will also include the positive control group in the follow‐up study. In conclusion, these findings suggest that SGP may offer therapeutic potential for the treatment of CTX‐induced liver injury.

## 5 Conclusions

In this study, the contents of total ginsenosides and minor ginsenosides were increased in sterilized fresh ginseng pulp, which promoted the transformation of major ginsenosides into minor ginsenosides. Additionally, the ginsenosides Rk2 and Rh3 were obtained, which were not present in the fresh ginseng pulp. In vitro antioxidant experiments indicated that the antioxidant activity of the fresh ginseng pulp increased following sterilization. We verified that SGP and FGP can alleviate CTX‐induced liver injury, which were responsible for the Nrf2 and MPAK signal pathways activation and apoptosis regulation. After treatment with SGP and FGP, the expression of the Nrf2 protein significantly increased, while that of the MPAK protein significantly decreased. This led to the accumulation of antioxidant enzymes and ultimately enhanced the antioxidant system, inhibiting oxidative damage. These results may offer a novel treatment option for mitigating the toxic side effects of CTX and could foster the development and application of fresh ginseng pulp. More importantly, it laid a foundation for developing fresh ginseng as a health food.

## Author Contributions

**Bo Nan:** funding acquisition (equal), writing – original draft (equal). **Guangquan Ren:** data curation (equal), formal analysis (equal). **Yutong Liu:** data curation (equal), formal analysis (equal). **Linqing Zhou:** software (equal). **Linlin Cui:** software (equal). **Jing Ge:** data curation (equal), software (equal). **Yidan Luo:** data curation (equal), software (equal). **Haihua Shi:** data curation (equal), software (equal). **Xia Li:** writing – review and editing (equal). **Yu Wang:** writing – review and editing (equal). **Yuhua Wang:** writing – review and editing (equal).

## Conflicts of Interest

The authors declare no conflicts of interest.

## Supporting information


**Table S1:** Monomer ginsenoside content.
**Figure S1:** The chromatographic profiles of FGP and SGP samples. (A‐C) FGP samples, (D‐F) SGP samples.

## Data Availability

The data supporting the findings of this study are available upon request from the corresponding author.
