# Supplementary material for: Components Changes in Fresh Ginseng Pulp Treated With Commercial Sterilization and Its Potential Therapeutic in CTX‐Induced Liver Injury via Apoptosis and Nrf2‐MAPKs/NF‐κB Pathways
Source: Food Sci Nutr. 2025 Nov 17;13(11):e71190. doi: 10.1002/fsn3.71190 (PMC12621000; doi:10.1002/fsn3.71190)
Supplement: Supplementary file 1 — Table S1: Monomer ginsenoside content. Figure S1: The chromatographic profiles of FGP and SGP samples. (A‐C) FGP samples, (D‐F) SGP samples. [file FSN3-13-e71190-s001.docx]

Supplementary Table 1. Monomer ginsenoside content.

| Monomer ginsenoside | FGP  Ginsenoside content (mg/g) | SGP  Ginsenoside content (mg/g) |
| --- | --- | --- |
| Ro | 3.339 ± 0.1164**^a^** | 0.391 ± 0.0234^b^ |
| Ra3 | 0.589 ± 0.0314**^a^** | 0.337 ± 0.0043^b^ |
| Rb1 | 0.668 ± 0.0236**^a^** | 0.630 ± 0.0192**^a^** |
| Rb2 | 0.948 ± 0.0227**^a^** | 0.497 ± 0.0031^b^ |
| Rb3 | 1.019 ± 0.0245**^a^** | 0.534 ± 0.0034^b^ |
| Rc | 1.031 ± 0.0247**^a^** | 0.540 ± 0.0034^b^ |
| Rd | 0.467 ± 0.0068**^a^** | 0.428 ± 0.0425**^a^** |
| Re | 0.220 ± 0.0091**^a^** | 0.214 ± 0.0260**^a^** |
| Rf | 0.701 ± 0.0272**^a^** | 0.587 ± 0.0246^b^ |
| F1 | 0.040 ± 0.0003**^a^** | 0.497 ± 0.0034^b^ |
| F2 | 0.124 ± 0.0071**^a^** | 0.328 ± 0.0087^b^ |
| F3 | 0.876 ± 0.0119**^a^** | 0.700 ± 0.0176^b^ |
| F11 | 0.695 ± 0.0270**^a^** | 0.582 ± 0.0244^b^ |
| Fe | 0.238 ± 0.0105**^a^** | 0.217 ± 0.0059**^a^** |
| Ft1 | 0.002 ± 0.0002**^a^** | 0.020 ± 0.0012^b^ |
| Rg1 | 0.413 ± 0.0645**^a^** | 0.372 ± 0.0128**^a^** |
| Rg2 | 0.069 ± 0.0039**^a^** | 0.097 ± 0.0030^b^ |
| Rg3 | 0.081 ± 0.0031**^a^** | 0.558 ± 0.0177^b^ |
| Rg5 | 0.011 ± 0.0002**^a^** | 0.878 ± 0.0304^b^ |
| Rg6 | 0.011 ± 0.0002**^a^** | 0.920 ± 0.0318^b^ |
| Rh1 | 1.205 ± 0.0751**^a^** | 1.097 ± 0.0212**^a^** |
| Rh2 | 0.002 ± 0.0001**^a^** | 0.064 ± 0.0023^b^ |
| Rh3 | 0.001 ± 0.0001**^a^** | 0.018 ± 0.0003^b^ |
| Rk1 | 0.058 ± 0.0010**^a^** | 0.917 ± 0.0220^b^ |
| Rk2 | 0.000 ± 0.0001**^a^** | 0.020 ± 0.0004^b^ |
| CK | 0.024 ± 0.0008**^a^** | 0.031 ± 0.0016^b^ |
| PPD | 0.075 ± 0.0012**^a^** | 0.072 ± 0.0012**^a^** |

Supplementary Figure 1.

A


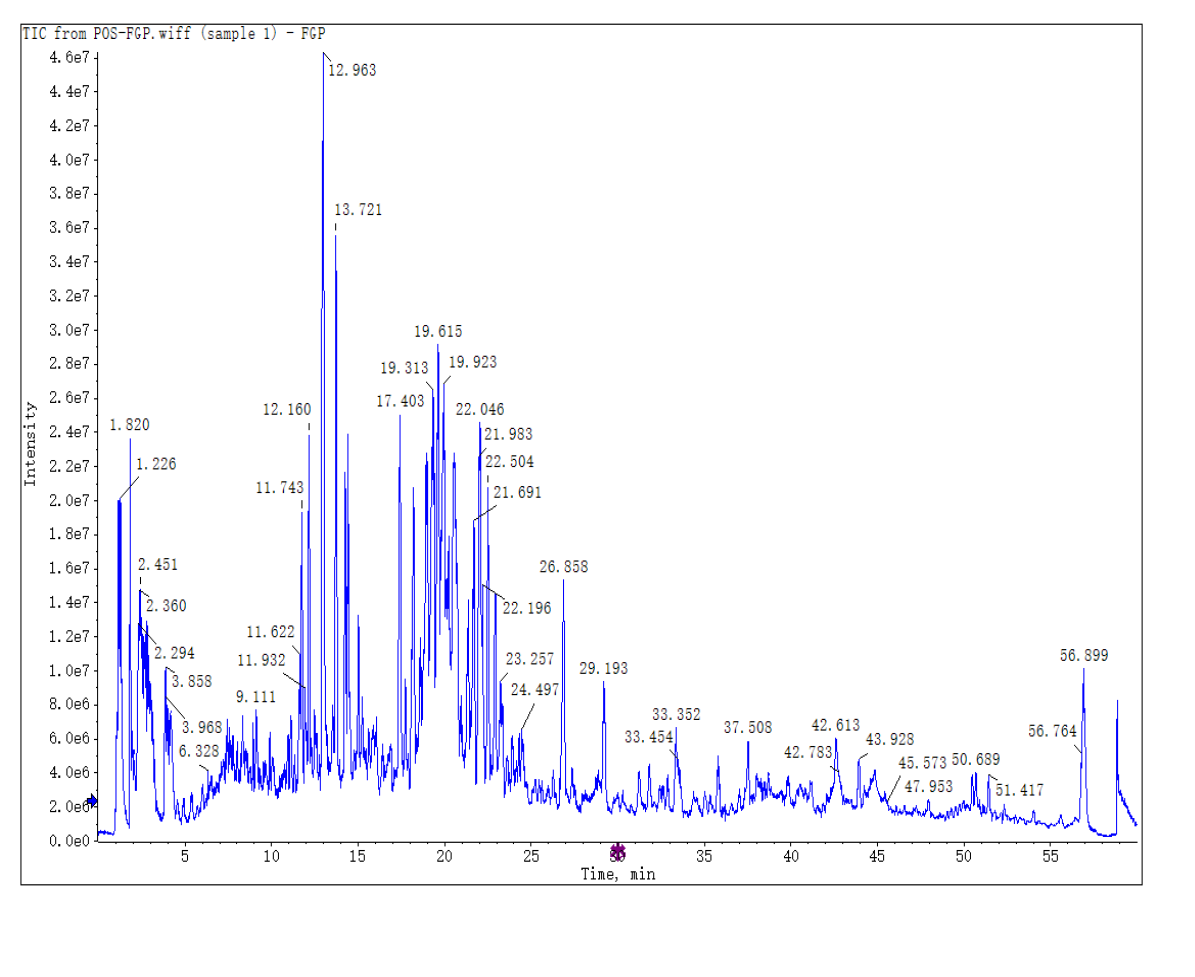


B


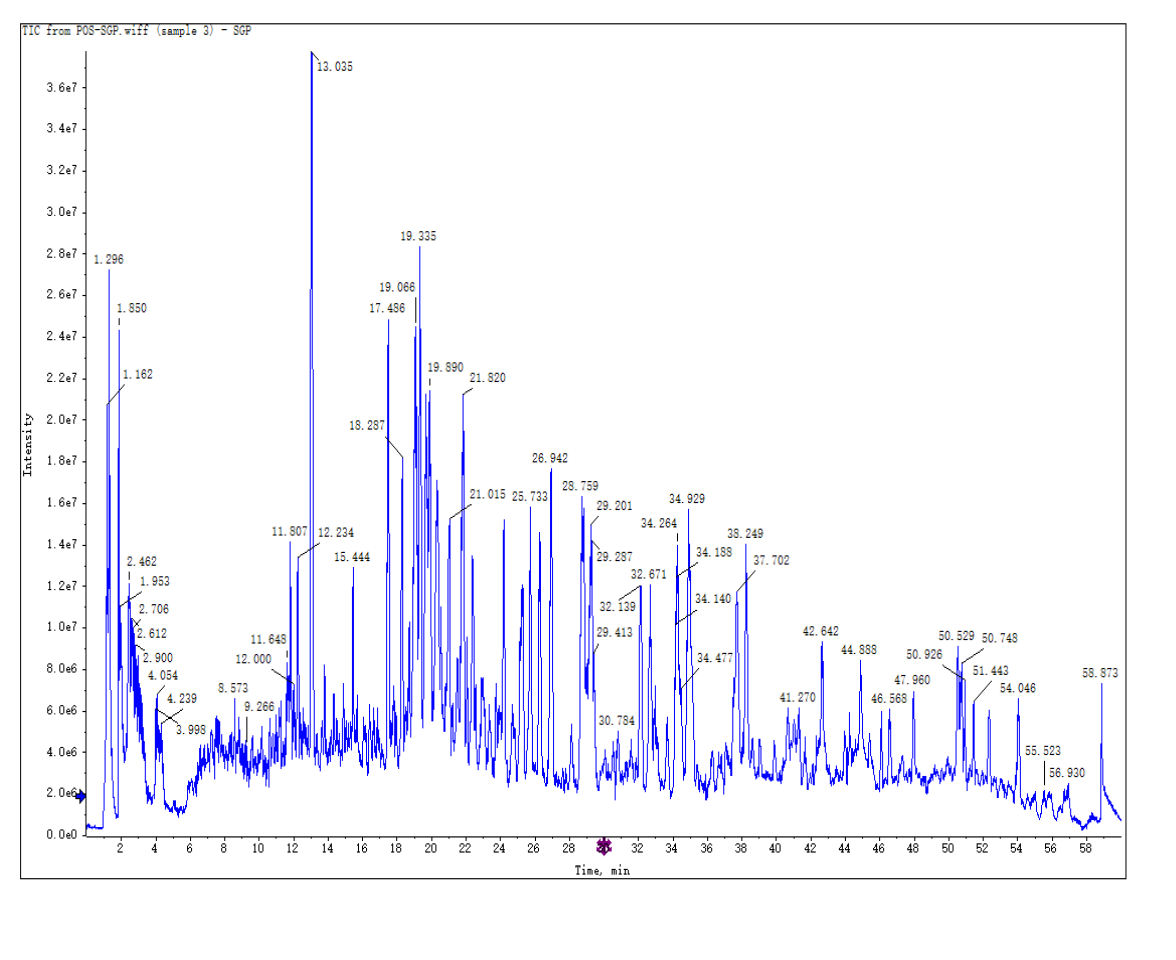


C


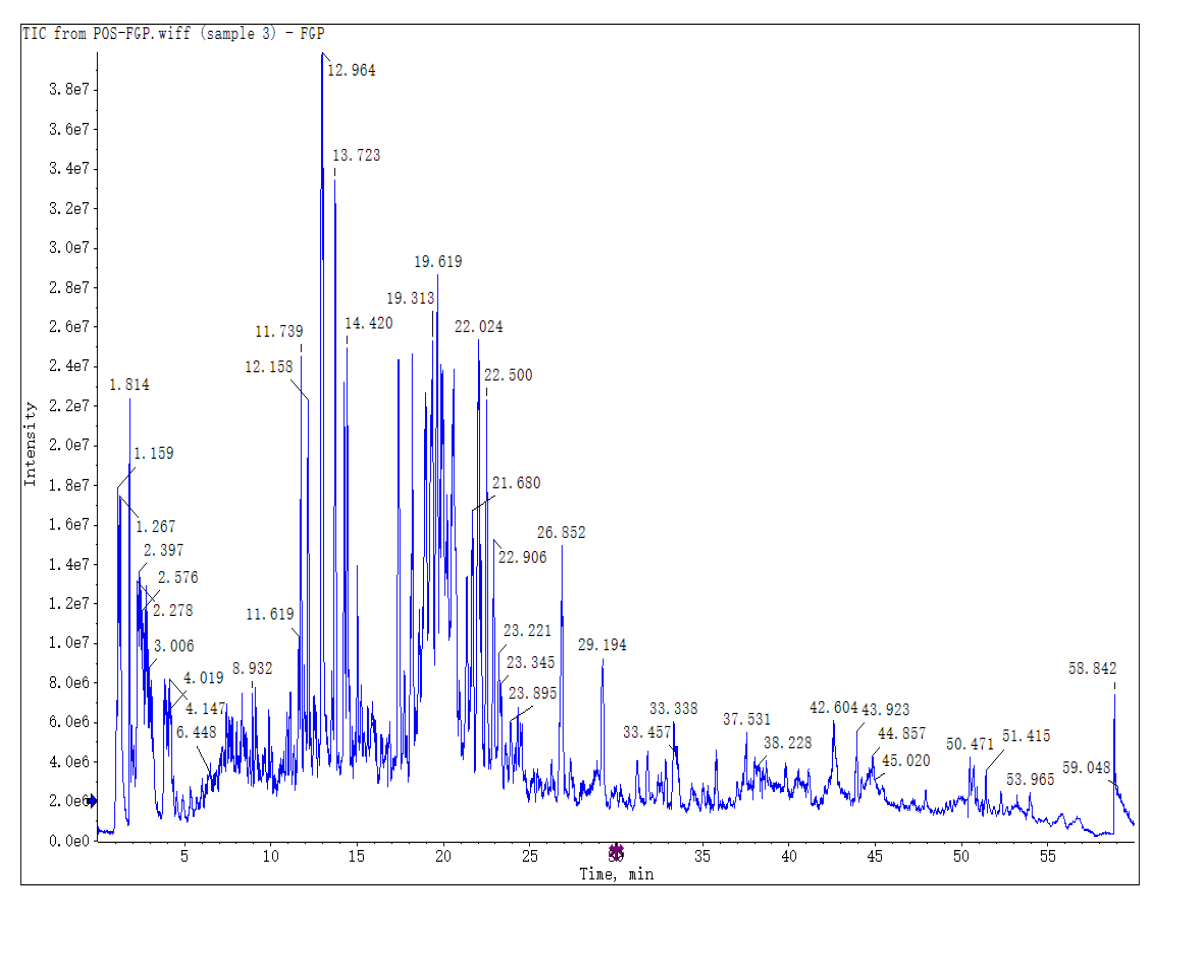


D


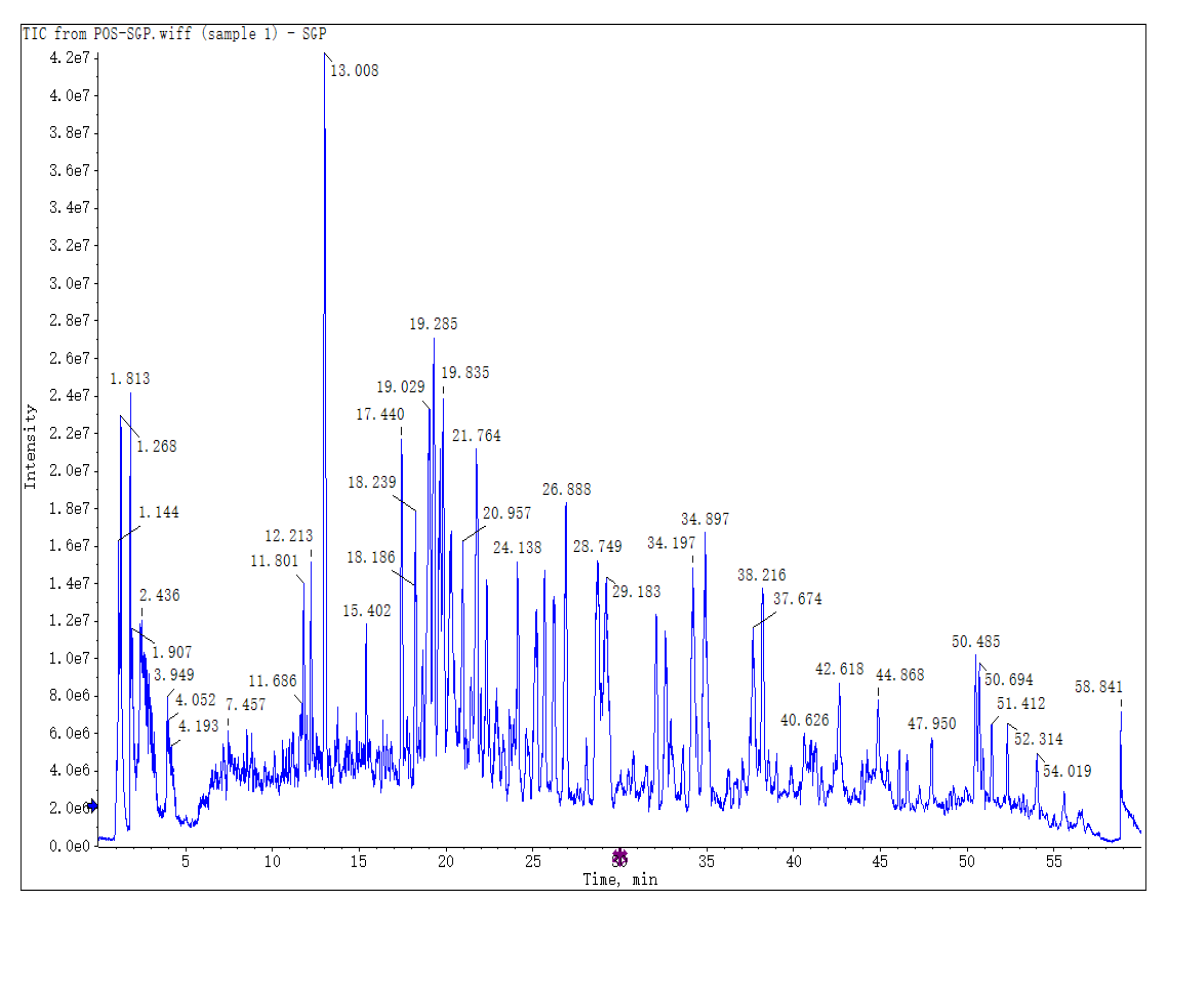


E


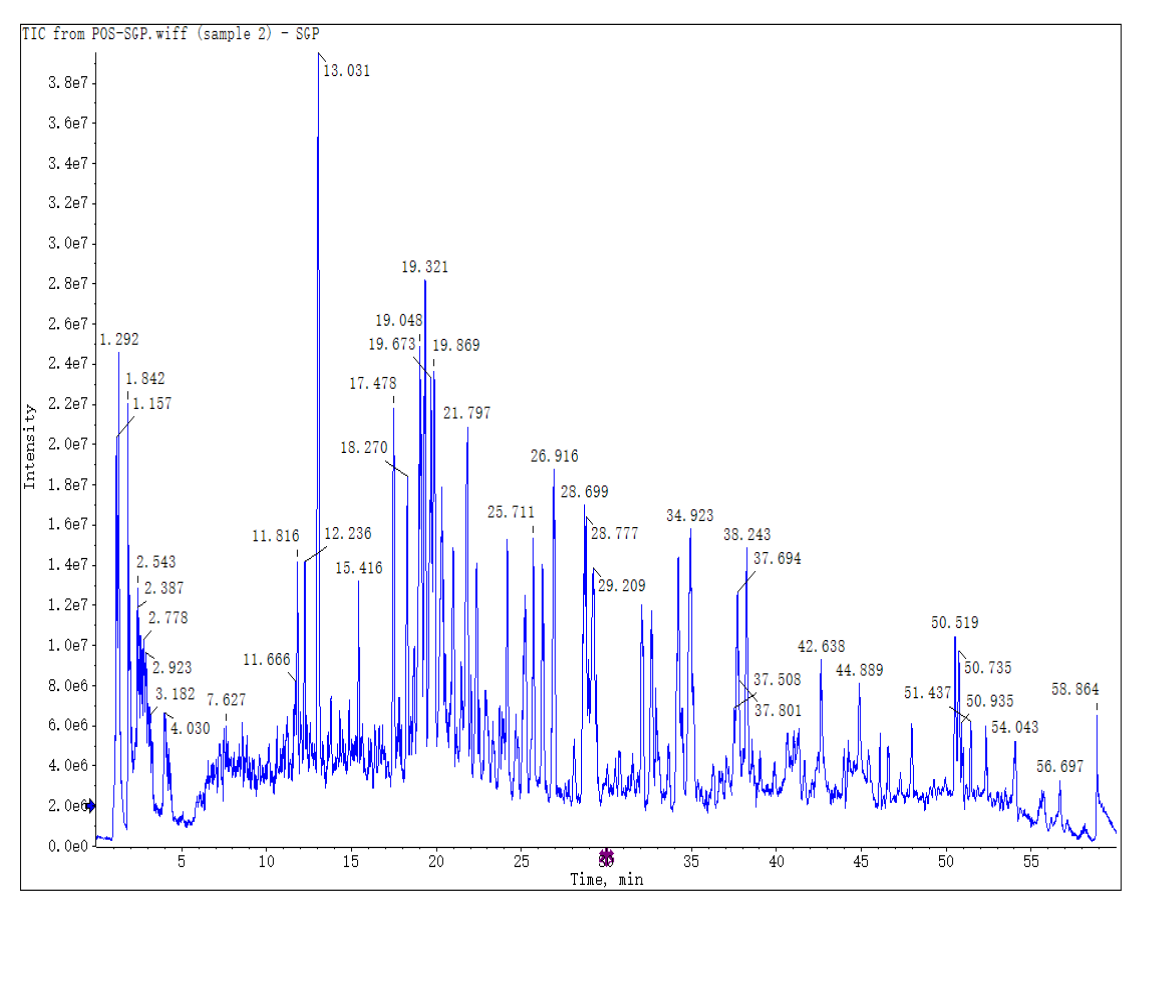


F


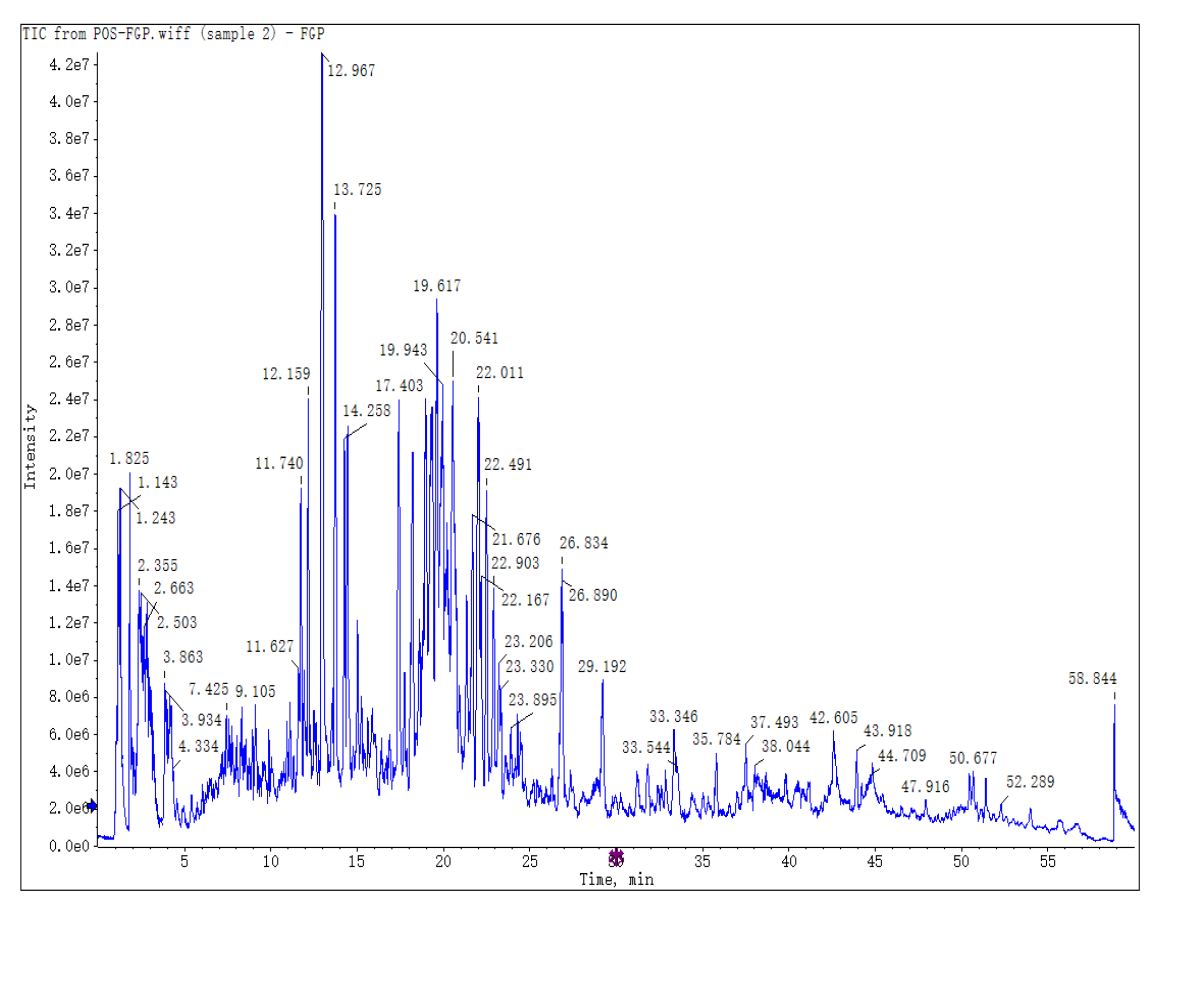


**Supplementary Figure 1.** The chromatographic profiles of FGP and SGP samples. (A-C) FGP samples, (D-F) SGP samples.
